# Transient Expression of Dengue Virus NS1 Antigen in *Nicotiana benthamiana* for Use as a Diagnostic Antigen

**DOI:** 10.3389/fpls.2019.01674

**Published:** 2020-01-16

**Authors:** Lívia É. C. Marques, Bruno B. Silva, Rosa Fireman Dutra, Eridan O. P. Tramontina Florean, Rima Menassa, Maria Izabel F. Guedes

**Affiliations:** ^1^ Laboratory of Biotechnology and Molecular Biology, Health Sciences Center, State University of Ceara, Fortaleza, Brazil; ^2^ Department of Biomedical Engineering, Biomedical Engineering Laboratory, Federal University of Pernambuco, Recife, Brazil; ^3^ Agriculture and Agri-Food Canada, London Research and Development Centre, London, ON, Canada

**Keywords:** non-structural protein 1, dengue, recombinant protein, elastin-like polypeptide, transient expression

## Abstract

Dengue is a viral disease that represents a significant threat to global public health since billions of people are now at risk of infection by this mosquito-borne virus. The implementation of extensive screening tests is indispensable to control this disease, and the Dengue virus non-structural protein 1 (NS1) is a promising antigen for the serological diagnosis of dengue fever. Plant-based systems can be a safe and cost-effective alternative for the production of dengue virus antigens. In this work, two strategies to produce the dengue NS1 protein in *Nicotiana benthamiana* leaves were evaluated: Targeting NS1 to five different subcellular compartments to assess the best subcellular organelle for the expression and accumulation of NS1, and the addition of elastin-like polypeptide (ELP) or hydrophobin (HFBI) fusion tags to NS1. The transiently expressed proteins in *N. benthamiana* were quantified by Western blot analysis. The NS1 fused to ELP and targeted to the ER (NS1 ELP-ER) showed the highest yield (445 mg/kg), approximately a forty-fold increase in accumulation levels compared to the non-fused protein (NS1-ER), representing the first example of transient expression of DENV NS1 in plant. We also demonstrated that NS1 ELP-ER was successfully recognized by a monoclonal anti-dengue virus NS1 glycoprotein antibody, and by sera from dengue virus-infected patients. Interestingly, it was found that transient production of NS1-ER and NS1 ELP-ER using vacuum infiltration of whole plants, which is easier to scale up, rather than syringe infiltration of leaves, greatly improved the accumulation of NS1 proteins. The generated plant made NS1, even without extensive purification, showed potential to be used for the development of the NS1 diagnostic tests in resource-limited areas where dengue is endemic.

## Introduction

The Dengue virus (DENV) is a positive-sense RNA virus belonging to the Flaviviridae family and to the genus Flavivirus. There are four distinct DENV serotypes (DENV1, DENV2, DENV3, and DENV4) and several genotypes within each of them (Roberts, 2015). The virus is the etiologic agent of dengue fever, a neglected disease which incidence has increased 30-fold over the last 50 years (Guzman and Harris, 2014).

Several factors drive this pandemic, including globalization, the spread of the *Aedes* mosquito vector, inadequately planned urbanization, and the absence of globally licensed vaccine or anti-dengue therapeutics (Simmons et al., 2012; Guzman and Harris, 2014). The Dengvaxia vaccine developed by Sanofi Pasteur was licensed in some countries, including Brazil, Mexico, the Philippines, Indonesia, Costa Rica, Paraguay, and El Salvador. However, it displayed low efficacy against serotype 2, and it has shown declined protection against DENV, especially in seronegative individuals (Hadinegoro et al., 2015; Halstead and Russell, 2016).

Despite an annual global incidence of 390 million cases, it is estimated that only 25% of the infected patients develop the clinical form of the disease (Bhatt et al., 2013). Inadequate access to sensitive and specific diagnostic tests contributes to the burden of the disease, therefore, innovative dengue diagnostic tests are needed for the management of clinical cases, epidemiological surveillance, and outbreak investigation (Peeling et al., 2010).

The DENV non-structural protein 1 (NS1) is a 46 to 50 kDa glycoprotein that can be bound to the endoplasmic reticulum (ER) membrane system of the infected cells (Flamand et al., 1999; Das et al., 2009). The NS1 monomer is modified by the addition of high-mannose carbohydrate moieties and rapidly assumes a dimeric presentation (dNS1), and then associates to the organelle's membranes. Subsequently, in the Golgi, other modifications in carbohydrate structures of NS1 dimers are added. A portion of NS1 traffics from the ER to the Golgi, where dimeric units associate to form soluble hexamers which are subsequently released from the infected cells (Muller and Young, 2013). High concentration of NS1 can be found in blood samples from infected individuals, from the onset of symptoms up to 9 days after the onset of the disease (Alcon et al., 2002).

Since the DENV NS1 can be detected before the establishment of an antibody response, this protein had been regarded as a potential biomarker for early diagnosis of dengue fever. However, the usefulness of this biomarker has been demonstrated only in primary infections. Subsequent infections with one of the three remaining DENV serotypes induce the rapid formation of NS1 immune-complexes, impairing the sensibility of diagnostic tests dependent on this protein. In this case, the serological detection of anti-NS1 IgM/IgG is recommended (Muller et al., 2017).

The immunoglobulin M-specific capture enzyme-linked immunosorbent assay (ELISA) (MAC-ELISA) is the standard serological test for the detection of anti-DENV IgM antibodies (WHO, 2009), and various dengue diagnostic tests are commercially available (Panbio Dengue IgM capture kit, Panbio Dengue duo cassette, Pathozyme dengue M capture and Dengue Vírus IgM Capture DxSelect™ EL1500M). These tests employ antigens produced by laborious and expensive methods, like immunopurified proteins from insect cells culture. These methods represent the major bottleneck for large-scale production of diagnostic tests for low-income countries (Gecchele et al., 2015; Amaro et al., 2015).

The production of recombinant NS1 protein in *Escherichia coli* often leads to insoluble, aggregated, or incorrectly folded products (Das et al., 2009; Sankar et al., 2013; Yohan et al., 2017; Chura-Chambi et al., 2019), which may compromise their utility in immunodiagnostic tests (Peternel et al., 2011). Different expression vectors have been developed to circumvent these limitations, including mammalian cells, insect cells, yeasts, and transgenic plants (Avirutnan et al., 2006; Nascimento et al., 2018; Altmann et al., 1999; Amaro et al., 2015; Thiemmeca et al, 2016; Allonso et al., 2019).

Transient expression of plant-derived viral antigens can be easily achieved by agroinfiltration (Mazalovska et al., 2017; Mbewana et al, 2018) and is preferential over stable transformation, saving material, and time spent for the generation of stably transformed plants (Marsian and Lomonossoff, 2016). The transient expression does not depend on chromosomal integration and is not affected by position effects (Komarova et al., 2010), making it suitable for rapid screening of a wide variety of different constructs (Marsian and Lomonossoff, 2016).

Compared to the other expression systems, the transient expression of recombinant proteins in plants offers many advantages such as reduced risk of contamination by human or animal pathogens. This system also allows eukaryotic-specific post-translational modifications of the produced proteins (Tschofen et al., 2016). The low level of intracellular accumulation of some recombinant proteins (<1% of the total soluble protein), and their purification have been the main bottlenecks of plant-made products (Martínez et al., 2010; Islam et al., 2018).

High levels of protein accumulation can be achieved by targeting the heterologous proteins to the appropriate subcellular compartment (Ahmad et al., 2010). The type of cell compartment in which they are found directly influences the folding, assembling, and posttranslational modifications processes, as well as preventing immediate degradation and interference of the polypeptides with cell metabolism (Ahmad et al., 2010; Leite et al., 2019). Typically, the main compartments to which recombinant proteins are targeted include the apoplast, the vacuole, the endoplasmic reticulum, the chloroplasts, and the cytosol (Pereira el at., 2014; Viegas et al., 2015; Miletic et al., 2016)

It has also been shown that adding fusion tags, such as elastin-like polypeptides (ELP), hydrophobins (HFBI), and Zera (an N-proline-rich region of gamma zein) to recombinant proteins increases their levels of accumulation. In general, ELP fusions increased the production yield of these target proteins by 2- to 100-fold (Conley et al., 2011) and HFBI fusion led to an increase of GFP accumulation in *Nicotiana benthamiana* leaves from 18% to 38% of TSP (Joensuu et al., 2010). These three protein fusion systems have also been shown to induce the formation of protein bodies, dense, and spherical structures derived from the ER, that protect the recombinant protein from the cell's molecular turnover, while also simplifying the process of recombinant protein purification by non-chromatographic methods (Conley et al., 2011).

The results obtained using the subcellular targeting strategies or fusion to insoluble tags depend on the nature of the recombinant protein (Viegas et al., 2015). Although other recombinant DENV proteins have been previously targeted to the ER (Seajung et al., 2007; Kim et al., 2009), these strategies have not been tested on the DENV NS1 accumulation in plants. Moreover, this study was the first to produce the DENV NS1 by transient expression in *N. benthamiana*.

To optimize the levels of DENV NS1 accumulation in *N. benthamiana* leaves, the DENV NS1 construct was targeted to five different subcellular compartments [apoplast, endoplasmic reticulum (ER), vacuole, chloroplast, and cytosol]. Then the construct with the highest accumulation level was fused to the ELP or HFBI tags. Our results show that when the KDEL ER-retention signal and the ELP partner were fused at the C-terminus of NS1, the highest level of protein accumulation was achieved. These strategies have been shown to be viable for large scale production of antigens since plant-produced NS1 was recognized by the sera of dengue positive patients, even without further purification.

## Materials and Methods

### NS1 Plant Expression Vectors

**Figure 1:**
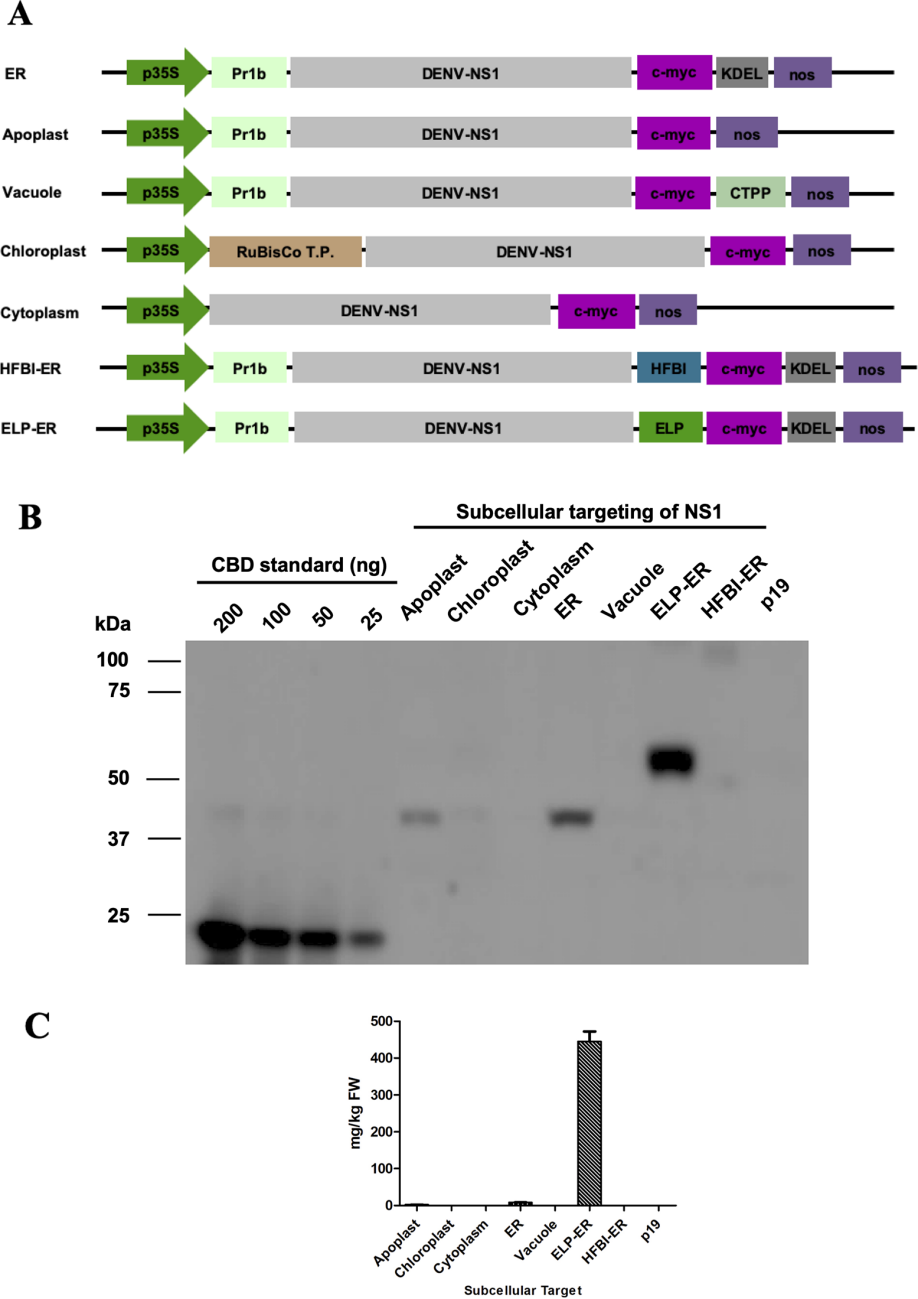
Subcellular targeting strategies tested for increasing accumulation of NS1 in *N. benthamiana* leaves. **(A)** Schematic representation of the DENV-NS1 expression constructs evaluated in this study: p35S, double enhanced 35S promoter from Cauliflower Mosaic Virus 35S gene; tCUP, translation enhancer from the tobacco cryptic upstream promoter; nos, nopaline synthase transcription terminator; Pr1b, tobacco pathogenesis-related 1b protein secretory signal peptide; C-Myc, detection/purification tag; KDEL, endoplasmic reticulum retrieval tetrapeptide; CTPP, vacuole sorting peptide; RuBisCo T.P., rubisco small subunit transit peptide; HFBI, hydrophobin I; ELP, elastin-like polypeptide. The schematic is not to scale. **(B)** Western blot analysis of NS1 targeting the protein to five different subcellular compartments in *N. benthamiana* leaves 4 days post-infiltration. Protein was extracted with PEB buffer, and an equal volume of TSP (20μl/lane) was loaded on the gel and detected with an anti-c-Myc antibody. Expression levels were measured by densitometry of western blot using known amounts of c-Myc-tagged CBD protein (25–200 ng) as reference; p19-infiltrated tissue was used as negative control. **(C)** Accumulation of NS1 and NS1 fusions in different subcellular compartments, results are the average of three experiments, and error bars represent the standard error of the mean.

The NS1 gene sequence from DENV serotype one was retrieved from Genbank (Accession number EF032592.1), and it was chemically synthesized (Biobasic Inc, Canada). The gene was cloned into a series of pCaMGate plant binary vectors (**Figure 1**) (Pereira et al., 2014) using the Gateway technology^®^ to generate constructs targeting five different compartments of the plant cell (apoplast, ER, vacuole, chloroplast, and cytosol). Two other ER constructs were developed for the expression of the NS1 protein fused to the HFBI or ELP tags (**Figure 1A**). All vectors included the coding region for the c-Myc tag that was used for protein detection. Each construct was transformed into the *Agrobacterium tumefaciens* strain EHA105, as described by Pereira et al. (2014).

### Transient Expression in *N. benthamiana*

*N. benthamiana* plants were grown in a growth chamber at 22°C with a 16-h photoperiod for 6 to 7 weeks. *Agrobacterium* cultures of each NS1 construct were combined with equal amounts of *Agrobacterium* culture containing the p19 suppressor of post-transcriptional gene silencing from Cymbidium Ringspot Virus (Silhavy et al., 2002), prepared as previously described (Saberianfar et al., 2015). For bench-scale expression, agroinfiltration of the abaxial leaf epidermis through the stomata of *N. benthamiana* plants was performed with a 1 ml syringe. All the constructs were infiltrated into at least three different experiments.

Large scale expression of NS1 ELP-ER construct was achieved by vacuum chamber infiltration. *Agrobacterium tumefaciens* cultures were grown to an optical density (OD600) of 1.0 to 2.0. Ten liters of *Agrobacterium* culture were mixed to 5 L of p19 culture and diluted with sterile distilled water to 30 L. The final volume was used for the vacuum infiltration of five trays (with thirty *N. benthamiana* plants each) into a sealed acrylic chamber connected to a VPD3 vacuum pump (VIOT, Champagne, U.S.A.). Vacuum was applied for 1 min (25 mm Hg) and slowly released to allow complete infiltration of the leaves. Plants were maintained in a controlled growth chamber for at least 4 days before harvesting.

### Protein Extraction

Different buffers were used for protein extraction. Initially, total soluble proteins (TSP) were extracted in ice-cold Phosphate Extraction Buffer (PEB) [phosphate-buffered saline (PBS)], pH 7.8, 0.1% (v/v) Tween-20, 2% (w/v) PVPP (polyvinyl polypyrrolidone), 1 mM EDTA (ethylenediaminetetraacetic acid), 100 mM sodium ascorbate, 1 mM PMSF (phenylmethylsulfonyl fluoride), and 1 μg/ml leupeptin. Afterward, Tween-20 was replaced by the same amount of Triton X-100 for the extraction of proteins for ELISA assays (PEB-Triton buffer).

For bench-scale infiltration, three leaf discs (7 mm diameter) from each plant were collected and frozen in liquid nitrogen. Each of these samples was pulverized using 2.3 mm ceramic beads in a TissueLyser (Qiagen) and resuspended in 200 μl of buffer. For the large-scale experiment, eight leaf discs from random plants in each tray were used for protein extraction. After grinding, 400 μl of buffer was added to each of these samples. Protein extraction from a plant infiltrated only with the p19 culture was also performed under similar conditions as a negative control. TSP concentration of all samples was quantified using the Bradford assay with bovine serum albumin as standard (Bradford, 1976).

An alternative buffer was used for the extraction of membrane bound proteins. For this, Tris Extraction Buffer (TEB) was used and consisted of 100 mM Tris-HCL pH 8.0, 1.5% (v/v) Triton X-100, 300 mM sucrose, 0.15 mM NaCl, 2% (w/v) PVPP, 1 mM EDTA, 20 mM DTT (dithiothreitol), 100 mM sodium ascorbate, 1 mM PMSF, and 1 μg/ml leupeptin.

### Inverse Transition Cycling With Ammonium Sulfate

After TSP extraction, the NS1 ELP-ER samples were submitted to Inverse Transition Cycling (ITC) for the purification of the elastin-tagged protein. Briefly, ammonium sulfate was added to an aliquot of the NS1 ELP-ER sample until a final concentration of 0.4, 0.6, 0.8, 1.0, or 1.2 M was achieved. The samples were incubated at 23°C or 37°C and then centrifuged (20,000 × g; 40 min). The supernatant 1 (S1) was collected, and the pellet was resuspended in PEB buffer to resolubilize the proteins, after which a new centrifugation was performed (20,000 × g; 4°C; 10 min). Soon after, the supernatant containing the solubilized protein (S2) was collected, and the remaining pellet was recovered in ice-cold 1× PBS buffer for analysis. After purification, the samples were analyzed by SDS-PAGE and Western Blotting, as described below.

### Western Blotting Analysis

TSP were separated by 10% SDS-PAGE, transferred to a PVDF membrane, and then incubated with mouse anti-c-Myc monoclonal antibody (Genscript, A00864, Piscataway, USA). ONE-HOUR Western Basic Kit Mouse (GeneScript, Piscataway, EUA) and Clarity Western ECL Substrate (Bio-Rad, Mississauga, Canada) were used to detect the recombinant proteins in a MicroChemi 4.2 (DNR Bio-Imaging Systems, Jerusalem, Israel). Western blots were analyzed using image densitometry with TotalLab TL100 software (Nonlinear Dynamics, Durhan, USA). The NS1 constructs were quantified by comparison with known amounts of a synthetic positive control protein containing a cellulose-binding domain (CBD) and a c-Myc tag (synthesized by Genscript, Piscataway, USA).

The molecular identity of the produced protein was further validated by western blotting analysis under non-reducing conditions against a specific antibody. Therefore, NS1 ELP-ER protein was purified by affinity chromatography using the C-Myc Tagged Protein Mild Purification Kit (MBL, 3305, Woburn, USA) according to the manufacturer's instructions. A monoclonal antibody against the full length native DENV NS1 glycoprotein (Abcam, ab41616) was used for protein detection (1:100 dilution).

### Serum Samples

For this study, sera from 27 DENV positive patients and sera from 27 DENV negative patients were obtained from the Central Laboratory of Public Health in the state of Ceara (LACEN/CE, Brazil), in 2013. The sera included positive MAC-ELISA IgM (Panbio, Australia) of all four DENV serotypes as well as positive DENV cases not serotyped from 2012 epidemics. For all analyses, the samples were fully anonymized. The Institutional Research Ethics Committee (CEP), State University of Ceara-Brazil, approved all experiments (approval 07520838-5/2009), which were carried out in accordance with the Declaration of Helsinki.

### NS1 ELP-ER ELISA for Anti-DENV NS1 IgM Detection

The usefulness of the produced protein as an antigen for dengue diagnosis was evaluated by an indirect ELISA using TSP from infiltrated plants. Thus, 10 μg of TSP was diluted in carbonate–bicarbonate buffer (pH 9.6) and incubated overnight at 4°C for the coating of 96 well MicroWell™ PolySorp^®^ plate (Sigma Aldrich, USA). The unbound protein was discarded and followed by five consecutive washes with PBS-Tween 80 0.05% (PBS-T) and then blocked with PBS with 5% non-fat milk (PBS-M) for 1h at 37°C. After the plates were incubated for 60 min at 37°C with 100 μl of patient sera diluted in PBS-M (1:100) and washed three times with PBS-T. The plates were incubated for 60 min at 37°C with anti-human IgM (μ-chain specific)—peroxidase antibody produced in goat (Sigma Aldrich, USA) (1:10.000) in PBS-M. Following three final washes with PBS-T and adding the TMB (3,3',5,5'-Tetramethylbenzidine) substrate (Thermo Fisher Scientific, USA) to develop the chromogenic signal, the plates were read at 650 nm using the Multi-mode reader Synergy 2 (Biotek, Vermont, USA).

The test's cut-off was defined as the average absorbance of the negative samples plus three times its standard deviation (Lardeux et al., 2016). Samples with absorbance equal to or below the cut-off value were qualified as negative ones, and then, the sensitivity and the specificity of the test were determined (Parikh et al., 2008). Briefly, all the 54 sera were divided into four groups, according to their results on the NS1-ER-ELP ELISA test: true positives (TP), false negatives (FN), false positives (FP), and true negatives (TN). Then, the sensitivity [Sensitivity = TP/(TP + FN)] and the specificity [Specificity = TN/(TN + FP)] of the test were calculated.

### Statistical Analysis

All analyses were performed using Prism version 5.0 (GraphPad Software, Inc, La Jolla, CA, USA) and Excel 2013 (Microsoft, Redmond, WA). Student's *t*-test was used to analyze means with statistical differences. All *p*-values <0.05 were considered as statistically significant.

## Results

This work describes a new strategy for plant production of the DENV NS1 protein for the development of new immunodiagnostic tests. The protein was targeted to five subcellular locations (apoplast, ER, vacuole, chloroplast, and cytosol), and the ER targeted construct was also fused to ELP or HFBI tags (**Figure 1A**). Each of the seven different constructs of NS1 were transiently expressed into the leaves of 6- to 7-week-old *N. benthamiana* plants also infiltrated with a construct containing the suppressor of gene silencing, p19 (Silhavy et al., 2002). The amount of each NS1 construct was quantified 4 days post infiltration (dpi) by western blotting using a monoclonal antibody against the C-terminus fused c-Myc tag (**Figure 1B**).

### Protein Expression of the DENV NS1 Constructs

**Figure 2:**
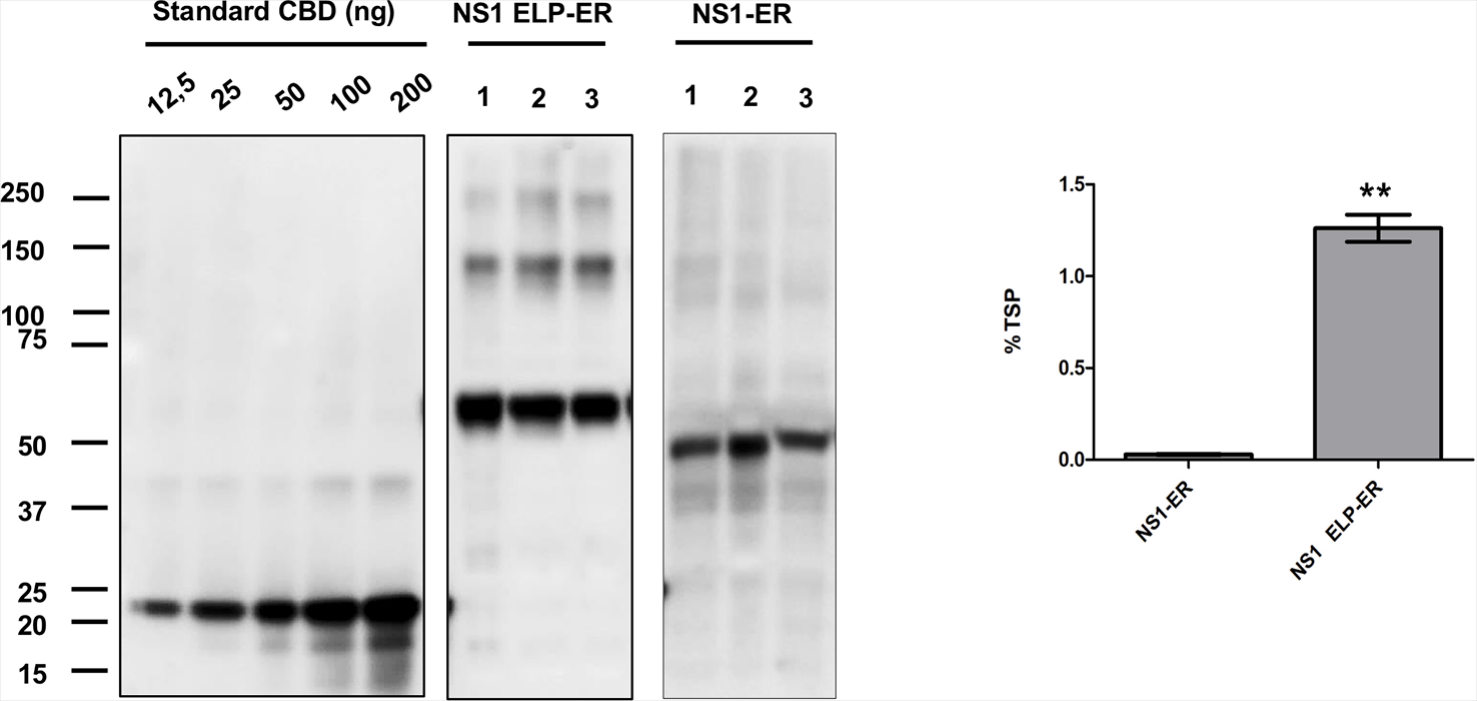
ELP fusion improves NS1-ER accumulation levels. *N. benthamiana* tissue was infiltrated with two different constructs, and the proteins were extracted with PEB buffer. Each lane was loaded with 20 μg of TSP from three replicates and was detected with anti c-Myc antibody. NS1 accumulation results represent the average of three biological replicates consisting of three different plants. Expression levels were measured by densitometry analysis using known amounts of c-Myc–tagged CBD protein (12.5–200 ng). Error bars are standard error of the mean (SEM). ^∗∗^Statistically significant difference by Student's *t*-test (*p* < 0.01).

After infiltration by syringe and protein extraction using the PEB buffer, it could be seen that the NS1 construct accumulated the most when targeted to the ER (8.18 ± 0.9 μg/g of fresh leaf weight), followed by the apoplast (2.19 ± 0.4 μg/g FLW). The protein did not accumulate to detectable levels when targeted to the vacuole, chloroplast, or cytosol of *N. benthamiana* leaves (**Figure 1**). The fusion of NS1 to ELP led to a high level of expression (445.2 μg/g of FLW), but no amount of NS1 could be detected when fused to HFBI (**Figures 1B, C**). The role of the ELP fusion over protein accumulation was validated by western blotting of three independent experiments of the constructs NS1-ER and NS1 ELP-ER, corroborating the difference of protein accumulation among these constructs (*p* < 0.01) (**Figure 2**).

**Figure 3:**
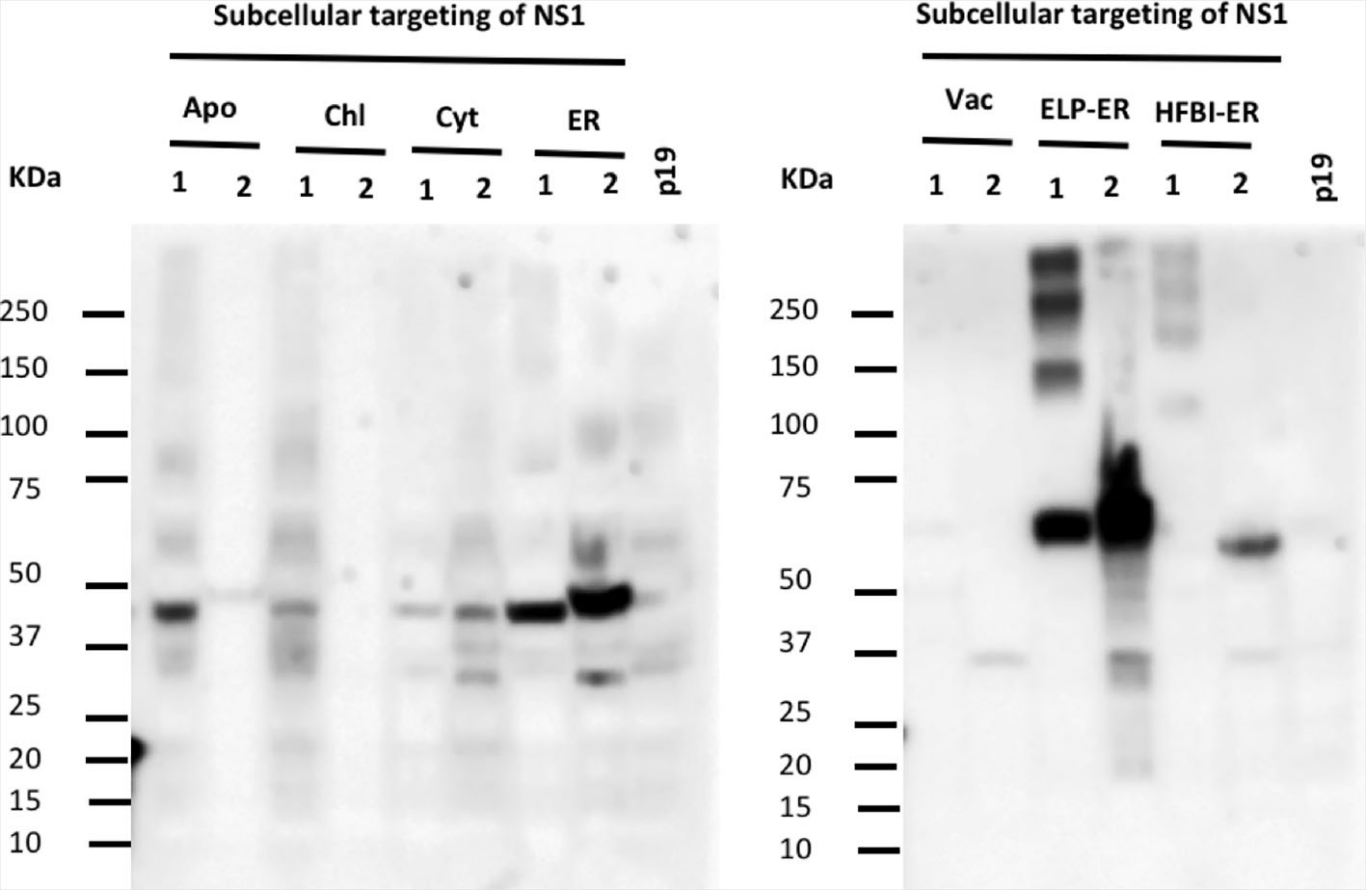
Triton X-100 improves extraction of NS1 from the ER. *N. benthamiana* leaves were infiltrated with seven constructs targeting the protein to different subcellular compartments. Proteins were extracted using two different buffers containing non-ionic detergents: 0.1% (v/v) Tween 20 (1) and 1.5% (v/v) Triton X-100 (2), the same volume of TSP (20μl/lane) was loaded per lane. The NS1 HFBI-ER construct showed protein accumulation only when extracted with Triton X-100 buffer.

Under reducing conditions, western blotting of proteins extracted with PEB buffer showed NS1 ELP-ER predominantly as a ~60 kDa protein, representing the monomeric form of NS1 48 kDa fused to ELP 11.5 kDa). Bands of higher molecular weight could also be seen, probably indicating the association of the protein into dimeric, trimeric, and tetrameric conformations (**Figures 2** and **3**).

Since no cytosolic, vacuolar, or chloroplast targeted construct could be detected by PEB buffer extraction, an alternative Tris-based buffer containing 1.5% (v/v) Triton X-100 (TEB) was used to improve NS1 extraction. The higher concentration of surfactant on TEB buffer resulted in a greater yield, mainly when the protein was targeted to the ER (**Figure 3**), but the proteins targeted to the other compartments could still not be detected. Furthermore, when TEB was used for NS1 HFBI-ER protein extraction, this construct could now be detected (48 μg/g fresh leaf tissue) (**Figure 3**).

When extracted on the TEB buffer, the NS1 ELP-ER multimeric forms were absent, resulting in a single 60 kDa band (**Figure 3**). After comparing the NS1 ELP-ER yield from PEB and TEB buffers, the latter yielded the highest amount of NS1 ELP-ER (1 mg/g fresh leaf tissue), being higher than the NS1 ELP-ER multimeric forms when combined (326 μg/g fresh leaf tissue) (**Figure 3**).

### Scale-Up and Purification of the NS1 ELP-ER Construct

Since the NS1-ER and NS1 ELP-ER constructs achieved the highest yields considering all the strategies used to this point, vacuum infiltration of plant trays was performed to test the scalability of NS1 production. After protein extraction with PEB, accumulation levels of 23.32 ± 5.15 and 639.6 ± 111.9 of fresh leaf tissue were observed for NS1-ER and NS1 ELP-ER, respectively (**Table 1**). There was an unexpected improvement of recombinant protein yield on the vacuum infiltration over syringe infiltration, and a comparison between the two methods is given in **Table 2**. Due to its higher yields, NS1 ELP-ER was the only construct chosen for the immunological assays.

**Table 1:** Transient expression of NS1 in *Nicotiana benthamiana* leaves using vacuum infiltration.

Replicate	NS1-ER		NS1 ELP-ER		
	**TSP %**	**μg/g FW**	**TSP %**	**μg/g FW**
1	0.0383	19.68	1.1499	741.16
2	0.0485	22.46	1.1548	768.43
3	0.0582	27.12	1.1362	612.31
4	0.0430	17.45	1.1593	507.85
5	0.0487	29.89	1.1279	568.19
Mean ± SD	0.0485 ± 0.007	23.32 ± 5.15	1.150 ± 0.01	639.6 ± 111.9

N. benthamiana leaves were vacuum-infiltrated to produce NS1-ER and NS1 ELP-ER. The leaves were collected four days post-infiltration. After protein extraction using PEB, the samples were assayed for NS1 by western blotting. The recombinant protein was quantified using TotalLab TL100 software. Data reported are means ± standard deviation (SD) from five independent replicates consisting of five different trays of infiltrated plants.

**Table 2:** Transient expression of NS1 in *Nicotiana benthamiana* leaves using syringe and vacuum infiltration.

	NS1-ER		NS1-ELP-ER	
Infiltration method	Syringe (μg/g FW)	Vacuum (μg/g FW)	Syringe (μg/g FW)	Vacuum (μg/g FW)
Mean ± SD	8.18 ± 0.9	23.32 ± 5.15	445.2 ± 47.0	639.6 ± 111.9

N. benthamiana leaves were infiltrated to produce NS1-ER and NS1 ELP-ER and then collected four days post-infiltration. The extracted proteins were assayed for NS1 by western blotting. The recombinant protein was quantified using Totallab TL100 software. Data reported are means ± standard deviation (SD) from independent replicates.

The ITC purification of the NS1 ELP-ER protein had shown that this protein could only be recovered at ammonium sulfate concentrations of 1.0 and 1.2 M (**Supplementary Figure 1**). At lower salt concentrations, no recovered protein could be detected in S2 (data not shown). The highest yield was obtained using 1.2 M ammonium sulfate at 23°C, with approximately 50% of NS1 ELP-ER recovered in fraction S2 (soluble) and the other half recovered in the pellet (**Supplementary Figure 1**). Despite of this, the recovered protein had shown low stability, leading to its irreversible precipitation short after its purification.

### Serological Analysis of the NS1 ELP-ER Construct

**Figure 4:**
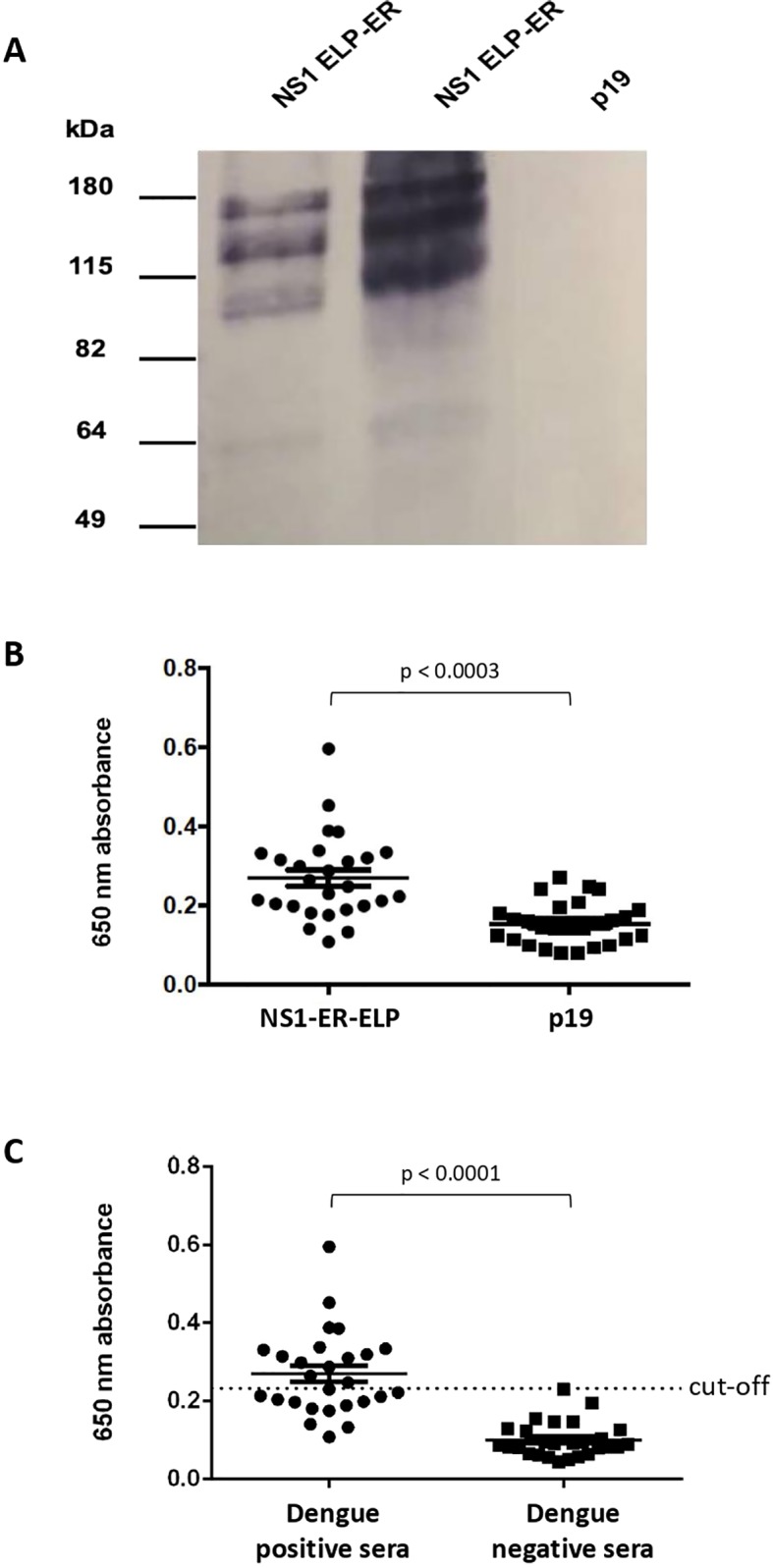
Plant produced NS1 as antigen for dengue diagnosis. **(A)** Recognition of NS1 ELP-ER produced in *N. benthamiana* by DENV anti-NS1 monoclonal antibody. NS1 ELP-ER was analyzed under non-reducing conditions. Immunoblotting with anti-NS1 monoclonal antibody against a conformational epitope revealed multimeric bands. p19-infiltrated leaves were used as negative control. **(B)** IgM–ELISA of the sera from 27 dengue positive patients against total soluble proteins from NS1 ELP-ER leaves or p19-infiltrated control (p19). The same samples were tested against each extract to evaluate the specificity of plant-produced NS1 ELP-ER. The means were significantly different as calculated by paired *t*-test, *p* < 0.0003. **(C)** NS1-ELP ELISA for IgM detection in dengue positive (n = 27) and negative (n = 27) sera. Unpaired *t*-test *p* < 0.0001. Mean value is shown by the thin line and the standard error of the mean (SEM) is represented by “whiskers.”.

The NS1 ELP-ER protein was then purified by immunoaffinity chromatography and separated by a non-reducing PAGE. The protein was recognized as a possible multimeric form by a monoclonal antibody that binds to a conformational epitope on the native NS1 protein. The predominant band detected was indicative of a dimeric form of the construct (~120 kDa). No equivalent to the NS1 monomers could be seen at the expected size of (~60 kDa) (**Figure 4**). This result demonstrates that plant-produced NS1 ELP-ER preserves (at least partially) the structure of the native protein and that its fusion to ELP does not interfere with NS1 folding or mask the analyzed epitope.

Lastly, to evaluate the NS1 ELP-ER potential for immunodiagnostics, an IgM ELISA screening test was performed. In this assay, 27 patient serum samples previously confirmed positive for dengue, and 27 dengue negative patient serum samples were used.

Due to the instability of the purified NS1 ELP-ER, microplates were coated with TSP from infiltrated and non-infiltrated *N. benthamiana* plants. The assay showed a significant difference between the mean absorbance of sera incubated with the protein extract from NS1 ELP-ER infiltrated leaves versus the protein extract from p19-infiltrated plants (**Figure 4B**). We also found that despite its low sensitivity of 48.15% (14 false negatives from 27 positive sera), the use of NS1 ELP-ER TSP for the immunoassay allowed the specific recognition of IgM from positive dengue sera (100% of specificity) (**Figure 4C**).

## Discussion

This work investigated the impact of targeting the recombinant DENV NS1 to plant subcellular compartments using specific signal peptides. Our results show that when the ER-retention signal KDEL was fused to the C-terminus of DENV NS1, the highest level of protein accumulation was achieved. This may reflect a prolonged interaction with ER-resident chaperones to promote correct folding (Benchabane et al., 2008), a stabilizing effect of glycosylation (Floss et al., 2009) or a limited proteolytic activity in the ER compartment (Vitale and Pedrazzini, 2005). On the other hand, the vacuolar and the chloroplast constructs could not be detected, highlighting the need for empirical tests for the choice of the proper compartment for recombinant protein production. Similar results have been previously described for other recombinant proteins expressed in *N. benthamiana* leaves. Miletic and colleagues (2016) described lower accumulation levels of Gp9 when targeted to the chloroplasts compared to the ER. Another report has shown that the subcellular accumulation of polygalacturonase was higher in the ER, followed by the apoplast and vacuole, while no detectable protein was found when targeted to the chloroplasts or cytosol (Pereira et al., 2014). Although some glycoproteins may accumulate well in the cytosol (Sainsbury et al., 2013) and chloroplasts (Tran et al., 2009), and perform the required biological functions, others such as NS1 could require glycosylation for stability and would not accumulate in those compartments. These analyses also demonstrated that the DENV NS1 recombinant protein expressed transiently in leaves accumulates to detectable levels especially when targeted to the secretory pathway. The ER is the first organelle of the secretory pathway and provides the most suitable environment for complex post-translational modifications, such as glycosylation and disulfide bond formation (Tschofen et al., 2016), Besides, ER targeting has been usually associated with enhanced recombinant protein yields in plants (Conley et al., 2009).

After the fusion of NS1-ER to the hydrophobin 1 from *Trichoderma reesei* (HFBI) or to the ELP, we found that ELP, but not HFBI, leads to a 40-fold increase in the yield of the recombinant product. The improved accumulation of the ELP fused protein could be due to an increased stability of the recombinant protein, either by reducing their susceptibility to hydrolysis or by packaging them into protein bodies (Conley et al., 2009). It has been shown that hydrophobins also increase protein accumulation when coupled to recombinant proteins through a similar protein body-inducing mechanism (Joensuu et al., 2010; Saberianfar et al., 2015). Although a previous study has demonstrated that the fusion to HFBI or ELP had similar effects over the accumulation level of the EspA protein from enterohemorrhagic *E. coli* (Miletic et al., 2017), the same effect could not be seen for the ER targeted DENV NS1. Our results corroborate the findings from Phan and colleagues (2014), which have shown that ELP, but not HFBI, fusion to hemagglutinin increases accumulation levels of the produced protein. These controversial data on the scientific literature highlight that recombinant constructs should be empirically tested before choosing the best fusion partner for each protein.

Our results have also shown that the elevated concentration of the detergent in TEB increased the amount of protein extracted (even when multimeric forms were considered in the quantification of NS1 ELP-ER) (**Figure 3**). With this buffer, the NS1 HFBI-ER could now be detected, leading to the hypothesis that the recombinant DENV NS1 protein could be bound to the ER membrane when transiently expressed in *N. benthamiana*. The production and purification of the Porcine epidemic diarrhea virus (PEDv) membrane protein by Khamis (2016) had shown that Triton X-100 was the most efficient detergent for extracting the *N. benthamiana* transiently expressed protein.

Plants have numerous advantages as a platform for the production of proteins used as diagnostic reagents, vaccines, and drugs, also called plant-made pharmaceuticals (PMPs) (Leite et al., 2019). This platform is easily scalable, requiring only soil, water, and light for the production of a large amount of biomass. The benefits include safety due to the absence of replicating human pathogens, and the speed of transient expression potentially providing gram quantities of product in less than 4 weeks (Buyel, 2019). The plant system is simple because sterility is not required during production, unlike classical expression systems, such as cell culture (Obembe et al., 2011). Although downstream processing costs are comparable to those of microbial and mammalian cells, the lower initial investment required for commercial production in plants and the potential economy of large-scale cultivation are the main advantages (Gecchele et al., 2015).

Our results indicate that agroinfiltration with both syringe and vacuum methods have resulted in the efficient expression of NS1-ER and NS1 ELP-ER. We found that transient production of NS1-ER and NS1 ELP-ER using vacuum infiltration of whole plants was easy to scale up, and there was an unexpected improvement of recombinant protein yield by using vacuum agroinfiltration compared to the use of a syringe. Huang et al. showed similar results with Hepatitis B surface antigen transiently expressed in *N. benthamiana* leaves infiltrated with the MagnICON viral vectors (Huang et al., 2008).

The transient expression of proteins in *N. benthamiana* for the diagnosis of other viral or neglected diseases has been already evaluated. Mbewana et al. (2018) demonstrated the potential of the plant produced Rift Valley fever virus (RVFV) nucleoprotein as an antigen for the development of a serological test, which differentiated control from the sera of RVFV-infected animals (Mbewana et al., 2018). Similarly, the Hepatitis E Virus (HEV) capsid protein expressed in *N. benthamiana* was also able to detect infection in both human and animal sera (Mazalovska et al., 2017).

In the current study, the expression of recombinant NS1 was scaled up by vacuum infiltration of whole plants, and it achieved yields as high as 639.6 μg/g of fresh leaf weight. Two different groups have used transgenic plants for the production of DENV antigens for the development of specific and inexpensive dengue fever diagnostic kits. The DENV2 NS1 and a tetra-epitope peptide of E protein from the four DENV serotypes were expressed in *Nicotiana tabacum* and lettuce chloroplasts, respectively (Maldaner et al., 2013; Amaro et al., 2015), but the yield of these recombinant constructs have not been described.

Several authors had proposed the use of prokaryotic systems for high-level expression of DENV NS1 (Das et al., 2009; Sankar et al., 2013; Yohan et al., 2017; Chura-Chambi et al., 2019). However, this system often leads to the loss of conformational epitopes, since it is well described that *E. coli* expressed proteins tend to precipitate under the form of inclusion bodies (Das et al., 2009; Sankar et al., 2013). Swan et al. (2018) compared plant (*N. benthamiana*) and bacterial (*E. coli*) produced G protein from the Nipah virus. They have reported that an anti-NiV antibody detected the plant produced protein, but not the same protein expressed in *E. coli*. The integrity of the produced NS1 ELP-ER protein was confirmed by immunoblot analysis in which dimers and other multimeric forms of this protein were recognized by a monoclonal antibody that binds to a conformational epitope on the native NS1. It indicates, thus, that the plant-produced antigen has structural and immunochemical characteristics similar to native DENV NS1.

The fusion to ELP was effective in increasing protein accumulation of plant produced DENV NS1. This happened without interfering with NS1 folding and assembly into dimers and multimers and without affecting its detection by patient sera. Several ELP fused proteins have been made in plants, and its activity was maintained despite the fusion to ELP. Floss et al. reported the fusion of an ELP to an anti-HIV-1 antibody for the treatment of HIV. They demonstrated that the fusion of 2F5 antibody's light and heavy chains to ELP did not impact the attachment of oligosaccharides to its glycosylation site, and that the glycosylation patterns as well as the kinetic binding parameters were identical to those of a CHO cell counterpart lacking ELP (Floss et al., 2008). Phan and Conrad (2011) demonstrated the successful fusion of ELP to two avian flu H5N1 antigens expressed in transgenic tobacco plants. They confirmed that the proteins retained their biological activity despite being fused to ELP (Phan and Conrad, 2011). Lastly, in 2016, Ikeda and colleagues developed a protein nanoparticle-based immunoassay to detect cancer biomarkers using a bioluminescent fusion protein, comprised of ELP with a poly-aspartic acid chain, NanoLuc^®^ luciferase, and a biotin acceptor peptide. The detection sensitivity for the cancer marker α-fetoprotein was found to be about ten times higher using ELP-D-Nluc-BAP nanoparticle-based ELISA compared to the assay using the monomer form of the protein enabling the construction of an ELISA with enhanced sensitivity (Ikeda et al., 2016).

Once fused to the ELP tag, the target protein should be readily purified by the ITC methodology (Despanie et al., 2016; Yeboah et al., 2016). However, this was not true for the NS1 ELP-ER construct, which had demonstrated low stability and then irreversibly precipitated along the process. The attempt to resolubilize the construct under chaotropic agents like 8 M urea or 6 M guanidinium thiocyanate solutions had also failed (data not shown). We speculate that characteristics intrinsic to the DENV NS1 protein (like its hydrophobic nature and the spontaneous association of multiple NS1 monomers) (Muller and Young, 2013) have contributed to its irreversible precipitation after recombinant expression.

Since NS1 ELP-ER reached 1.15% of TSP and it could still be detected by an antibody against a DENV NS1 conformational epitope (**Figure 4A**), we sought to evaluate the usefulness of the TSP of infiltrated leaves as a substrate for dengue fever diagnosis. The dengue virus NS1 ELP-ER recombinant protein was able to detect IgM antibodies against DENV NS1 without the need for extensive purification, which could probably reduce the production costs of the diagnostic test, surpassing the major bottleneck of protein purification (Buyel, 2019).

The high concentration of contaminant proteins on the NS1 ELP-ER TSP have been shown to be the major drawback of the test, since host proteins became a steric hindrance for the NS1 ELP-ER coating of the plates, lowering the sensitivity of the test. Although the fusion to the ELP should allow at least the enrichment of the protein fraction, our attempts of protocol optimization had been unsuccessful. Our results on the use of plant TSP for DENV NS1 immunodiagnosis corroborates the work of Amaro et al. (2015), which used a crude extract from transgenic *N. tabacum* expressing DENV NS1, as a substrate for ELISA. The authors were able to differentiate between dengue positive and negative sera, but a second assay (sandwich ELISA) was needed to improve their results (Amaro et al., 2015).

The unique properties of ELPs have made them a desirable class of fusion tags used in several biomedical applications. The use of ELP as a fusion partner to NS1 enhanced the expression of this antigen; however, it did not allow its purification from plant extracts. The specific recognition of NS1 ELP-ER by the IgM antibodies of DENV infected patients even without purification reinforces the potential of plant produced NS1 protein for dengue diagnosis. However, a different strategy for protein purification would favor the large-scale production of dengue recombinant antigens for serodiagnosis.

## Data Availability Statement

The datasets generated for this study are available on request to the corresponding author.

## Ethics Statement

The Institutional Research Ethics Committee (CEP), State University of Ceara-Brazil, approved all experiments (approval 07520838-5/2009), which were carried out in accordance with the Declaration of Helsinki.

## Author Contributions

LM, MG, and EF conceived and developed the work plan. LM designed the research, performed the experiments, and wrote the manuscript. RM was an external co-supervisor to LM and involved in project planning and troubleshooting and contributed to writing. BS helped in plant-related experiments. LM, MG, RD, BS, EF, and RM edited the manuscript.

## Conflict of Interest

The authors declare that the research was conducted in the absence of any commercial or financial relationships that could be construed as a potential conflict of interest.
