# Supplementary material for: Transient Expression of Dengue Virus NS1 Antigen in Nicotiana benthamiana for Use as a Diagnostic Antigen
Source: Front Plant Sci. 2020 Jan 16;10:1674. doi: 10.3389/fpls.2019.01674 (PMC6976532; doi:10.3389/fpls.2019.01674)
Supplement: Supplementary file 1 [file Presentation_1.pptx]

## Slide 1
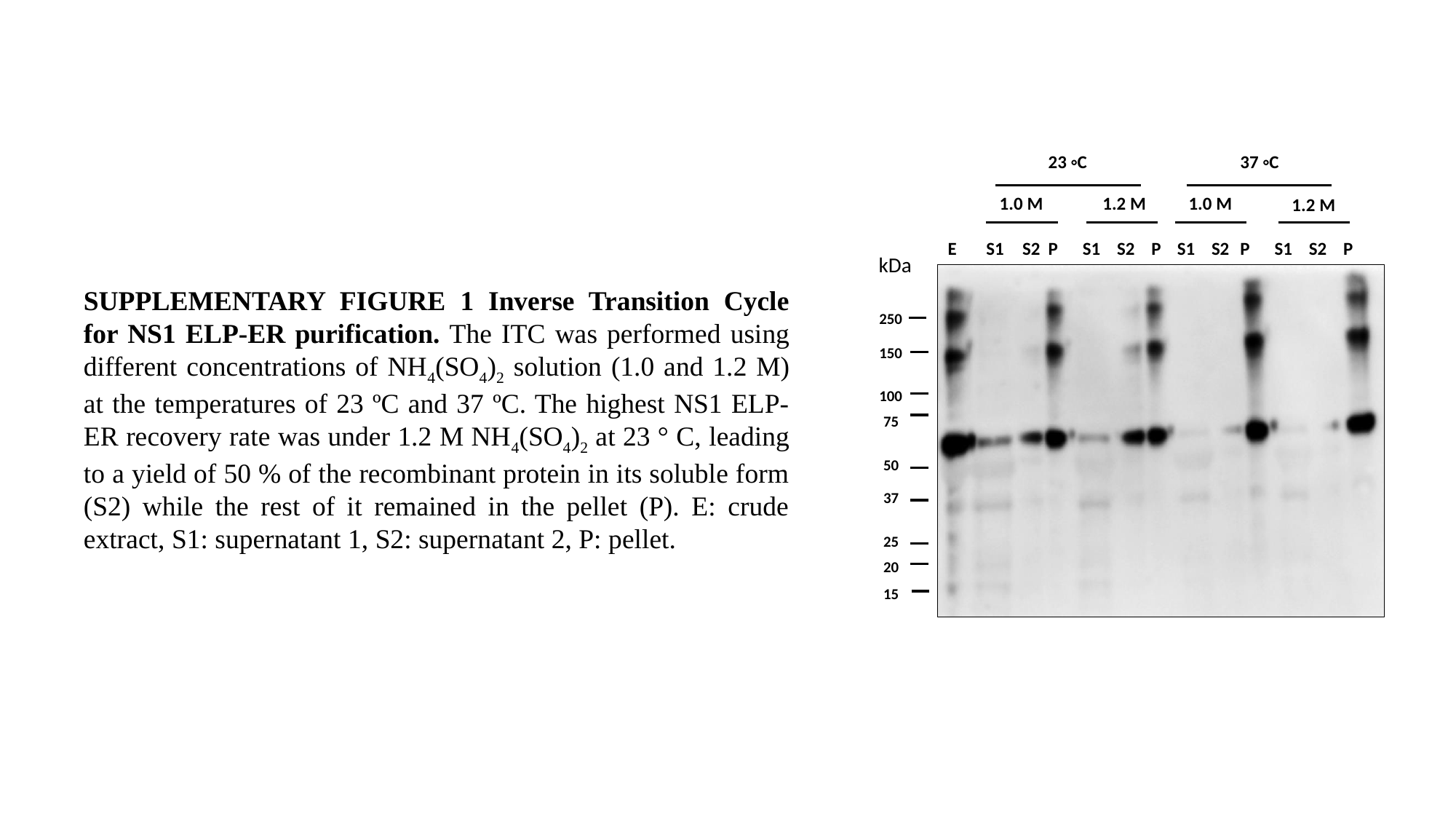

23 ◦C
37 ◦C
1.0 M
1.2 M
1.0 M
1.2 M
E
S1
S2
P
S1
S2
P
S1
S2
P
S1
S2
P
250
150
100
75
50
37
25
20
15
kDa
SUPPLEMENTARY FIGURE 1 Inverse Transition Cycle for NS1 ELP-ER purification. The ITC was performed using different concentrations of NH4(SO4)2 solution (1.0 and 1.2 M) at the temperatures of 23 ºC and 37 ºC. The highest NS1 ELP-ER recovery rate was under 1.2 M NH4(SO4)2 at 23 ° C, leading to a yield of 50 % of the recombinant protein in its soluble form (S2) while the rest of it remained in the pellet (P). E: crude extract, S1: supernatant 1, S2: supernatant 2, P: pellet.
